# Dietitians' perspectives on key components relevant for successful dietetic treatment of adults with obesity in primary health care: a qualitative study in the Netherlands

**DOI:** 10.1111/jhn.13387

**Published:** 2024-11-25

**Authors:** Annemieke van de Riet, Rebecca S. Otte, Harriët Jager‐Wittenaar, Marian A. E. de van der Schueren, Elke Naumann, the Dietetics Building the Future consortium, Kirsten Berk, Harriët Jager‐Wittenaar, Hinke Kruizenga, Jacqueline Langius, Barbara van der Meij, Elke Naumann, Marieke Plas, Annemieke van de Riet, Marian de van der Schueren

**Affiliations:** ^1^ Division of Human Nutrition and Health Wageningen University and Research Wageningen the Netherlands; ^2^ Research Group Nutrition, Dietetics, and Lifestyle HAN University of Applied Sciences Nijmegen the Netherlands; ^3^ Research Group Healthy Ageing, Allied Health Care and Nursing Hanze University of Applied Sciences Groningen the Netherlands; ^4^ Department of Gastroenterology and Hepatology Dietetics Radboud university medical center Nijmegen the Netherlands; ^5^ Department Physiotherapy and Human Anatomy, Research Unit Experimental Anatomy Faculty of Physical Education and Physiotherapy, Vrije Universiteit Brussel Brussels Belgium; ^6^ Erasmus Medical Centre, Department of Internal Medicine, division of Dietetics Rotterdam The Netherlands; ^7^ Hanze University of Applied Sciences, Research Group Healthy Ageing, Allied Health Care and Nursing Groningen The Netherlands; ^8^ Radboud university medical center, Department of Gastroenterology and Hepatology, Dietetics Nijmegen The Netherlands; ^9^ Vrije Universiteit Brussel, Faculty of Physical Education and Physiotherapy, Department Physiotherapy and Human Anatomy, Research Unit Experimental Anatomy Brussels Belgium; ^10^ Amsterdam UMC, Department of Nutrition and Dietetics Amsterdam The Netherlands; ^11^ Amsterdam Movement Sciences, Ageing & Vitality Amsterdam The Netherlands; ^12^ The Hague University of Applied Sciences, Faculty of Health, Nutrition and Sport, Department of Nutrition and Dietetics and Research group Rehabilitation and Technology The Hague The Netherlands; ^13^ Wageningen University and Research, Human Nutrition and Health, Research group Global Nutrition Wageningen the Netherlands; ^14^ HAN University of Applied Sciences, Research Group Nutrition, Dietetics, and Lifestyle Nijmegen The Netherlands; ^15^ HAN University of Applied Sciences, Research Group Nutrition, Dietetics, and Lifestyle Nijmegen The Netherlands; ^16^ Dutch Association of Dietitians Amersfoort The Netherlands; ^17^ Wageningen University and Research, Human Nutrition and Health, Research group Global Nutrition Wageningen the Netherlands; ^18^ Wageningen University and Research, Human Nutrition and Health, Research group Global Nutrition Wageningen the Netherlands; ^19^ HAN University of Applied Sciences, Research Group Nutrition, Dietetics, and Lifestyle Nijmegen The Netherlands

**Keywords:** counselling, dietetics, dietitian, obesity

## Abstract

**Background:**

Dietetic treatment of adults with obesity can result in effective weight loss with health improvements. However, it remains unclear which components of dietetic consultation are key for successful treatment of individual patients. Therefore, the aim of this study is to explore dietitians' perceptions of key components relevant for successful dietetic treatment of adults with obesity in primary health care in the Netherlands.

**Methods:**

In this phenomenological study, semistructured interviews were conducted with 18 dietitians who have experience in treating adults with obesity in primary care. Validation of interview data was performed through two focus group discussions with 14 dietitians. Thematic analysis was used to analyse the data.

**Results:**

Four main themes were identified: (i) building a good relationship; (ii) identifying patient needs; (iii) supporting behaviour change and (iv) providing advice. Dietitians highlighted the relevance of building a good relationship with their patients and emphasised adopting a counselling role alongside their role of educator. They also recommended the use of educational materials, counselling techniques and behaviour change strategies (e.g. goal setting, self‐monitoring, addressing barriers) to address specific patient needs, such as health literacy, self‐efficacy and motivation.

**Conclusions:**

This study demonstrates that dietitians perceive the ability to build a trusted relationship, in which patient needs are properly explored and addressed, as the key to successful dietetic treatment of adults with obesity. Our findings emphasise the importance of the dietitian's approach in this process and show that patient factors influence the choice of appropriate treatment approaches.

## Key points

- Establishing a trusted relationship with patients is key to successful treatment, enabling better exploration and addressing of patient needs.
- Tailoring dietetic treatment involves aligning strategies with individual patient factors, including motivation, self‐efficacy and (health) literacy.
- To support patients' lifestyle changes, more emphasis is needed on the dietitian's coaching role, offering encouragement and implementing behaviour change techniques.

## INTRODUCTION

Globally, the prevalence of obesity has doubled since 1990, with 16% of all adults being obese in 2022.^1^ The same trend is observed in the Netherlands, where 14% of the adult population was obese in 2021.^2^ Obesity contributes to a variety of chronic diseases and is associated with negative effects on mental health.^3^, ^4^, ^5^ A weight reduction of 5%–10% in people living with overweight or obesity has been associated with positive health outcomes on comorbidities, such as a reduction in fasting glycaemia, hemoglobin A1c, systolic and diastolic blood pressure and plasma lipid profile.^6^ Therefore, healthcare interventions for people with obesity are usually aimed at weight loss. A systematic review demonstrated that weight loss interventions provided by dietitians during controlled trials are effective in reducing body mass index, waist circumference and blood pressure and have a positive effect on quality of life compared to a control group usual care.^7^ However, in dietetic practice in the Netherlands, only a quarter of patients with overweight and obesity achieved weight loss of 5% or more and many patients drop out before the recommended treatment time of 6–12 months.^8^ Thus, dietetic treatment is not effective for a large group of patients, and yet, the underlying causes of this remain inadequately understood.^9^

Because the treatment of obesity is complex and requires a multimodal approach,^10^ dietetic treatment guidelines include a wide range of components that dietitians focus on in consultations with patients with obesity. These guidelines are mainly focused on diet characteristics and weight loss and include general guidance on counselling techniques and multidisciplinary cooperation.^11^ A review of dietetic guidelines from several European countries on the treatment of patients with obesity showed that it remains unclear which components of dietetic consultation are relevant for the successful treatment of individual patients.^12^ Dietitians combine the various recommended treatment components in individual treatment based on their experience, but it is unclear how different treatment components interact with each other.^13^ In contrast to the high number of studies focused on diet characteristics for maximising health benefits, the methods used by dietitians to facilitate these dietary shifts and lifestyle improvement in patients with obesity have been understudied. In addition, most research focuses on the outcomes of an intervention, rather than delving into the experiences of patients and dietitians or the dynamics of the patient–dietitian relationship.^14^ Previous studies report that treatment should focus on a positive patient–dietitian relationship, individualised care and patient involvement in their care process. However, guidance on how to tailor dietetic treatment based on different patient needs is lacking.

Insights into the experiences of practising dietitians are crucial for obtaining a more comprehensive understanding of the factors that contribute to treatment effectiveness and adherence. Therefore, the aim of this phenomenological qualitative study is to identify key components relevant for the successful dietetic treatment of patients with obesity from the perspective of primary healthcare dietitians in the Netherlands.

## METHODS

This study is part of the four‐year research project entitled ‘Dietetics building the future’ (Box 1). Data were collected through a multimethod qualitative study design, consisting of semistructured interviews, followed by two focus group sessions with primary care dietitians treating adults with obesity and comorbidities in the Netherlands. We reported the findings according to the Standards for Reporting Qualitative Research^16^ checklist (see Supporting Information).

Box 1. **Description of research project Dietetics building the future**

The research project ‘Dietetics building the future’ is a research collaboration of (applied) universities, practising dietitians and other relevant stakeholders in the Netherlands to develop an evidence‐based toolbox for personalised dietetic care for patients with obesity and co‐morbidities. The project addresses research questions regarding the optimisation of treatment, improving effectiveness and patients' self‐efficacy. Furthermore, the research pays special attention to patients with low health literacy, stimulating behavioural change and application of innovative technologies. The project consists of quantitative analyses and statistical modelling to identify key components of dietetic care. Concurrently, qualitative studies are conducted, such as the current study from the perspectives of dietitians, along with semistructured interviews with patients, to capture their experiences, needs and opinions. The results of the earlier research phases will be used for the development (co‐design) and testing of a toolbox, offering personalised interventions based on various individual patient factors. A summary of the project can be found on the website of ZonMw, the organisation that funded this research.^15^

### Participants and data collection

#### Individual interviews

In the Netherlands, dietitians operate within a two‐tier system comprising primary and secondary care. Primary care dietitians work in the community setting, providing nutritional counselling and interventions for chronic conditions such as obesity. Patients can visit primary care dietitians on their own initiative or can be referred by the general practitioner for either weight loss or dietary advice for comorbidities. Secondary care dietitians, on the other hand, are based in hospitals, where they manage more complex clinical cases, including malnutrition, metabolic disorders and pre‐ and postsurgical nutritional support. One‐on‐one interviews were conducted with primary care dietitians treating patients with obesity and comorbidities in the Netherlands. Semistructured interviews were used because they offer the opportunity to obtain rich data and contextual insights instead of aiming for generalisability.^17^ By using semistructured one‐on‐one interviews, we provided dietitians with an open space to share their thoughts and experiences. The interviews took place between November 2022 and September 2023.

We recruited dietitians through diverse channels, including the distribution of informational flyers in the newsletter of the Dutch Association of Dietitians and the LinkedIn page of Dietetic Building the Future Research Project (Box 1). To ensure that we captured a wide range of perspectives, we aimed to interview at least 16 dietitians. The sample size, is comparable to similar qualitative studies conducted in this field.^17^ Purposeful sampling was applied to achieve a heterogeneous study sample. To include dietitians treating patients with diverse characteristics, a preregistration questionnaire was used to gather information about the dietitians and their patient populations.

In the prescreening questionnaire, questions regarding dietitians' characteristics covered their age, years of professional experience, type of practice, including self‐employed and those employed in a dietitians practice, and geographical location. To ensure that participating dietitians worked with a diverse range of patients, we also used the prescreening questionnaire to select dietitians who represented various patient categories. These categories included patients with varying comorbidities, socioeconomic positions, migrant backgrounds, health literacy levels and places of residence (rural or urban). Special attention was given to dietitians treating patients with low (health) literacy levels.

A topic guide was created based on the literature on dietetic treatment of patients with obesity. This guide helped to provide consistency across the different interviews, ensuring that all relevant areas were covered. Additionally, it allowed participants to discuss topics important to them, even if these were not initially included in the guide, thus offering flexibility for in‐depth exploration of participant responses.^18^ The topic guide included the following topics: duration and frequency of consultations, experiences with successful or nonsuccessful treatment, treatment characteristics (e.g., conversation styles used, goal setting, providing feedback, tracking motivation, self‐monitoring), patient characteristics (e.g., comorbidities, health literacy), patient–dietitian relationships, behaviour change, information provision, new technologies, early drop‐out of patients, treatment outcomes and needs for tools to use in dietetic treatment. For each topic, several probing questions were predefined, but researchers could decide themselves whether to use these. One pilot interview was conducted with both first authors present to test the topic list and probing questions, after which some minor changes were implemented.

The interviews were conducted in Dutch by the primary authors of this research, RO and AvdR, who both have a background and expertise in the fields of human nutrition, health sciences and/or dietetics. The face‐to‐face interviews took place at the participants' dietetic practices, except for one, which was conducted at the participant's home upon their request. Conducting the interviews in the practice/home setting made it easier to build rapport, create a comfortable environment for the participants and observe social and nonverbal cues.^19^ Participants were informed about the aim and procedure of the interview and provided consent before the start of the interview. We aimed to build rapport between the interviewer and the participant by ensuring sufficient time for an introduction of the study and the interviewer and by highlighting that the interview is an open conversation in which there are no right or wrong answers.

Interviews lasted approximately one to one‐and‐a‐half hours. The first two interviews were conducted with both first authors (RO and AvdR) present, aiming to ensure a consistent approach throughout and to enable real‐time feedback. Dietitians received a 50‐euro voucher as reimbursement for their participation in the interviews. After completing the interviews and transcripts, member checks were conducted by providing three participants with summaries of their individual interviews. With these member checks, participants had the opportunity to review the summaries, provide feedback on the interpretations or findings and confirm their agreement with the representations of their perspectives and experiences.^20^ The participants agreed with the summaries and only minor additions were shared by participants via email.

#### Focus groups

Subsequently, focus groups were organised to enable dietitians to freely discuss topics raised in interviews with their peers. The use of focus groups alongside individual interviews provides a dual‐method approach that offers several advantages. First, it allows for a more comprehensive understanding of the subject through data triangulation, where insights from interviews are validated by the findings of the focus group discussion. Second, topics that may not have been extensively covered or remained unclear in interviews can be further explored and clarified in focus groups. Finally, the group setting of focus groups promotes interaction among the participating dietitians, which results in rich discussions and thereby deeper, and sometimes even new, insights into the topics.^21^ In addition to data triangulation, practical insights were gathered in the focus group discussions on how to translate important treatment components to practical tools to use in dietetic practice. This will be further explored in the next phase of the research in which a toolbox for dietitian will be developed (Box 1). Recruitment of dietitians for the focus groups was conducted in the same manner as for the interviews. For the focus groups, only dietitians who did not participate in the interviews were selected to obtain different perspectives. The focus group discussions took place in a conference room in two different cities, both located centrally within the Netherlands (Amersfoort and Almere), allowing dietitians from different regions to attend. We aimed to include six to eight participants in each focus group discussion. This number is large enough to allow diversity in the group and to facilitate a discussion. It also ensures that all participants have the opportunity to share, preventing the formation of subgroups.^22^ The sessions were scheduled during the evening, each lasting 2 h, with a 15‐min refreshment break. Moderation of the focus groups was carried out by the primary authors, RO and AvdR, with assistance from researchers from the Division of Human Nutrition and Health of the Wageningen University and Research. Participants were seated around a table and were encouraged to actively contribute to the discussions. All focus group participants provided informed consent before the start of the discussion. The conversations followed a predefined topic guide, serving as a guide for the discussion and allowing for a participant‐driven exploration. The participants had room to shape the discussion in their own direction, whereas the moderator ensured that all topics were covered. Topics for the focus groups were derived from the data analysis of the interviews to enable data triangulation and validate the findings from the interviews. To design the focus group topics, the two first authors (RO and AvdR) discussed the themes derived from the interviews, composed a list of topics and defined the main priorities. Together with two other authors (EN and HJ), the topic guide and protocol were finalised. The main topics were client characteristics, barriers and facilitators in treatment and motivation and behaviour change.

### Recording and transcription

All interviews and focus groups were recorded using audiorecording equipment and transcribed verbatim using Amberscript software. After the interviews and focus groups, the transcribed content was cross‐checked against the audio recordings and refined by fourth‐year BSc students in Dietetics engaged in the study to ensure the accuracy of the transcription.

### Data analysis

Data analysis of the interviews was conducted first, followed by the analysis of the focus groups. For both the analysis of the interview and focus group data, we used a thematic analysis approach as outlined by Braun and Clarke.^23^, ^24^ We used a stepwise approach for conducting thematic analysis by following their six‐step framework.

#### Familiarising with the data

Transcripts were read by the researchers to become familiar with the content of the interviews and to make intermediate adjustments in the topic list.

#### Generating initial codes

In the second phase, initial codes were applied to the interview data in the program ATLAS.ti Web (version 5.20.0). To ensure consistency in the coding process, both first authors (RO and AvdR) independently coded the first three interviews. Thereafter, the codes were discussed in a consensus meeting by RO, AvdR, EN and HJ. Subsequent interviews were coded separately by the two first authors and structural meetings were held between them to discuss the coding process and unclear codes. Coding was performed using both deductive and inductive methods. Due to the semistructured interview set‐up, codes deductively emerged from the preset topics. Additionally, because of the open nature of the interviews, new topics emerged, leading to inductive coding as well. The same principles used for the analysis of the interview data applied to coding the focus group discussions. The topic guide for the focus group discussions included the main topics from the interviews, leading to deductive coding, whereas the new topics that arose during the discussions were coded inductively.

#### Searching for themes

After coding all interviews, initial codes were grouped into preliminary themes. The collation of codes took place during a work session in which the two first authors (RO and AvdR) and a research assistant. In this session they discussed the relationships between all codes and emerging themes, and grouped and regrouped these into initial themes. The focus group discussions followed the same themes as the interviews.

#### Reviewing themes

In the next phase, the initial themes and subthemes from the interviews were discussed and assessed by RO, AvdR, EN and HJ, to determine whether the data that belonged to these themes cohered meaningfully and whether themes were adequately distinct from each other. This involved reviewing whether the coded extracts from the interviews fitted well within the themes, as well as assessing whether the themes reflected the content and meaning of the data set as a whole.

#### Refining and defining themes

After mapping of themes within the interview data, we further refined and defined the themes. This process included identifying subthemes and providing structure to the complex data set. In addition, we assigned appropriate titles to the themes to accurately reflect their content. During the theme refinement process, multiple discussion meetings were arranged between members of the research team.

#### Presenting the findings

The final step was to present the thematic analysis in a concise and comprehensive way, while providing sufficient evidence and examples of the different themes. In the presentation of the results, we aimed to represent the views of all participants, supported by individual statements. We reviewed data extracts within each theme to determine which fragments captured the overall message within each theme best. The Dutch quotes were translated into English and checked by a native speaker. Furthermore, the quotes were checked for personal details to ensure the anonymity of individual statements.

## RESULTS

### Participant characteristics

From the 35 dietitians who applied for the interviews, 18 dietitians (one male and 17 females) were selected to participate in one‐on‐one semistructured interviews, and in total, 14 dietitians participated in two focus group discussions from the 21 who applied. The selection was made based on the answers in the preregistration questionnaire and the targeted number of participants (at least 16 for interviews and six to eight for each focus group discussion). We conducted 18 interviews including the pilot interview. All participants were employed in primary care at the time of the study and treated patients with obesity, with or without comorbidities. They either worked as independent dietitians or within a larger dietetic organisation (salaried). The participants represented 10 out of the 12 provinces in the Netherlands and worked in both rural and urban areas. The youngest participant had worked less than five years as a dietitian, whereas the oldest had worked as a dietitian for more than 40 years.

### Themes

**Figure 1:**
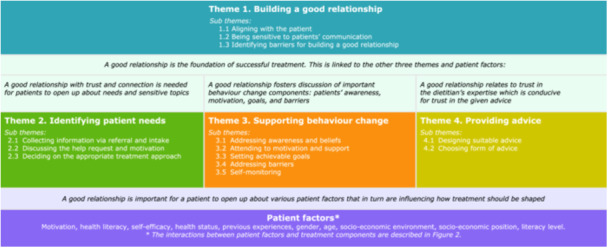
Overview of themes, subthemes and patient factors.

The interviews and focus group discussions covered a wide range of topics related to what treatment components are important for successful treatment. Successful treatment was defined and perceived by dietitians as achieving individually determined patient treatment goals and patient satisfaction. We identified four themes (Figure 1): (1) building a good relationship, (2) identifying patients' needs, (3) supporting behaviour change and (4) providing advice. Each theme consists of several subthemes that are presented as subheadings in the text.

**Figure 2:**
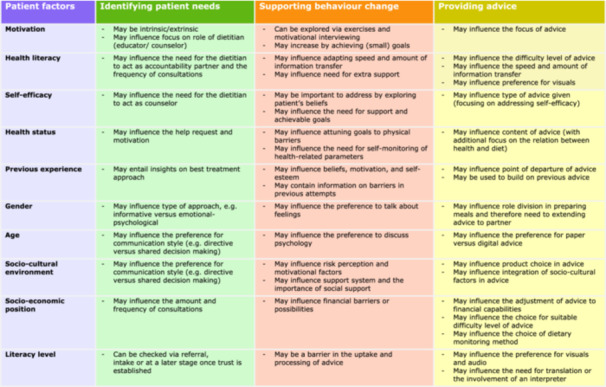
Matrix of patient factors that affect dietetic treatment with respect to identifying patients' needs, supporting behaviour change and providing advice, according to dietitians.

In addition to treatment components, several patient factors were distinguished by dietitians that interact with the various treatment components, as captured in Figure 2. Participants shared that although they sometimes notice patterns in patient factors, generalisation cannot and should not be made, as every patient is unique. Therefore, the points of attention for each patient factor related to the themes in Figure 2 are intended to provide general guidance for futher exploration, rather than strict criteria. The patient factors are integrated throughout the text rather than discussed separately; however, this matrix illustrates their importance across at every phase of treatment and across all themes.

## THEME 1: BUILDING A GOOD RELATIONSHIP

Participants perceived the interpersonal relationship between dietitian and patient to be the foundation and often a prerequisite for successful treatment.

Super important, super important. My focus is on the relationship. First, I need to feel that that is good and then we can continue. For me that is fundamental. Very occasionally I think: it doesn't work. Well I can tell you [if the relationship is not good]: the whole treatment will not work.

(Dietitian 18, interview)

Participants perceived that a good relationship forms the foundation for successful treatment and is linked to the other themes (Figure 2). First, having a good connection is pivotal in identifying patient needs (Theme 2), as a certain level of trust is needed for patients to open up, according to participants. When the patient requires the dietitian to adopt a counselling role in which emotions and psychology are discussed, the quality of the relationship was said to be highly important. On the other hand, when a patient predominantly seeks information, the interpersonal relationship was perceived to be of less importance. Second, to support behaviour change (Theme 3), patients need to feel safe and comfortable to share barriers, emotions,values and motivations that underly their behaviour, according to participants. Finally, participants shared that the relationship is important in providing advice (Theme 4), as they experience that patients tend to take the given information more seriously when a good relationship has been established with trust in the expertise of the dietitian.

### Aligning with the patient

The first contact was viewed as an important starting point for gathering information about the patient and building the therapeutic relationship. It was pointed out that a moment of contact even prior to the first visit, by a short phone call for example, may help provide familiarity and take away possible anxiety to overcome ‘the hurdle’ of a first visit. Participants shared that sharing their own vulnerabilities and using humour in the conversation or talking about light‐hearted topics before addressing treatment topics, can help in the process to make the patient feel at ease and build rapport. Participants mentioned several success factors for building a strong interpersonal relationship. They stated that it is more important to remain open and receptive to the patient—eliminating one's own opinion and not discussing differences in beliefs. An important factor in building the relationship was connecting with a patient over common interests or characteristics, such as both being dog owners, speaking the same dialect or sharing other similarities. Participants reported that one can always find something to connect over; the key is to display openness, respect and interest in the patients' situation and perceptions.

Then I tell them: I know a bit how rice dishes work [because] I'm from [that continent] myself. You notice that they get happy as their expectation was very low. I only say the word ‘okra’ [and] they know: ‘Alright, we can just be transparent about our habits’, you feel the client's relief, you feel a ‘she knows what she is talking about’.

(Dietitian 18, interview)

### Establishing trust

Participants indicated that once trust is established in the relationship, they feel that patients become more open and willing to share personal details about their circumstances.

In itself, a personal connection is not necessarily essential, depending on the nature of the issue. For example, with emotional eating, people already feel vulnerable or judged in advance. Well, in that case, that personal connection really helps them to further open up. Well, the tissues are here, they are used a lot; there's quite a bit of crying happening here. So, I think it's important for people to feel safe enough.

(Dietitian 10, interview)

Participants stated that being able to be vulnerable themselves can help to build trust, by, for example, sharing personal difficulties to make clear that everyone fights their own struggle. In addition, being honest and having an open, egalitarian attitude without judgement were found to be helpful towards building a good relationship.

### Being sensitive to patients' communication

In building a connection, participants emphasised the importance of being sensitive to the individual traits of the patient to build understanding, trust and collaboration. They mentioned that dietitians should have attentive ‘antennas’ that enable them to pick up on nonverbal cues and verbal expressions. Based on these notions, participants shared that they adapt their communication to the patient by tailoring the amount of detail, language complexity and tone. Developing these ‘antennas’ often comes with experience according to participants in our study. Another enabler was communicating on a first‐name basis and using informal forms of address.

I think as a dietitian, you just need to be able to sense things. I mean, in any kind of communication with people, you do. But you just try to sense things. Like, how is someone reacting to the conversation? And sometimes you just let people share their stories. And also gently steer them back towards the direction you want to go as a healthcare provider.

(Dietitian 9, interview)

### Identifying barriers for building a good relationship

Participants indicated that building a strong personal relationship can be challenging at times. For example, differences in interest and communication styles, as well as instinctive sense of liking or disliking the other, were reported as obstacles. In addition, language barriers or the presence of an interpreter can be a barrier for building a good relationship. Identifying one's own personal preferences and annoyances can help to be aware of underlying causes of not feeling a personal connection, according to participants. Participants shared that they can often sense in early stages of the treatment whether there is a genuine connection. In situations where the interpersonal connection is difficult or lacking, indicated that it may be more effective for the patient's treatment to refer them to another dietitian.

I sometimes have patients that I really don't feel a connection with. And then I tell them: well, maybe it is sensible that I will plan a consultation for you with my colleague, who can [better] help you further.

(Dietitian 3, interview)

## THEME 2: IDENTIFYING PATIENT NEEDS

Participants emphasised that properly getting to know the patient and their needs is pivotal in shaping successful dietetic treatment. From participants' perceptions, it is important to learn about a variety of patient factors (Figure 2) to tailor the patient's individual treatment trajectory.

To take into account the needs and desires of the person in front of you, instead of a standard list. Yes, that is, I believe, for me the most important.

(Dietitian 1, interview)

### Collecting information via referral and intake

Multiple ways were shared by participants for getting to know the patient. First, contact with other health professionals, such as the general practitioner in case of referral, was considered to be a valuable way of gathering general information about the patient even before the first consultation, for instance about the patient's health status and literacy level. Second, participants reported that they use the first consultation and sometimes a questionnaire to learn more about the patient's background. Finally, participants noted that the first consultation serves as an important moment to discuss expectations and possible prejudices, barriers or anxiety related to the dietetic treatment. During this session, a wide variety of topics are often discussed, such as the patient's daily life and eating habits, his or her social–cultural environment, socioeconomic position, health status, previous experiences, treatment demands, and motivation. Participants shared that patients with different backgrounds might need different focuses during the first consultation. For example, a more thorough in‐depth dietary assessment may be required for patients whose cultural background differs from that of the dietitian, according to participants. They noted that discussion of sensitive topics may be more appropriate to address at a later stage. For example, in the treatment of patients with a low literacy level, participants pointed out the importance of building trust, as in their experience, it takes time for patients to open up about having limited literacy skills. In addition, participants shared that simple tests can be used to assess the patient's literacy level. Finally, discussing expectations about treatment and, if necessary, redirecting these were viewed to be important.

And I think what really often hinders people is having incorrect expectations regarding the pace of weight loss. And yes, I believe that I always make it very clear during the intake what a responsible and realistic pace is in general.

(Dietitian 15, interview)

### Discussing the help request and motivation

According to participants, it is essential to discuss expectations of the treatment and to define treatment goals, preferably together with the patient. They emphasised that the ‘help request’ (referring to the patient's reason for seeking support) should determine the treatment goal.

Your [the dietitian's] directive is of course the patient's treatment demand. What does your patient hope to achieve? That is what you go along with. Also, when someone does not really have a treatment demand or is referred, then you investigate with the right questions: you are here now, where do you want to go? What is your direction? So maybe not a very concrete goal, but a general direction. That can also be enough. So, yes, the patient leads, and we [dietitians] follow.

(Dietitian 9, interview)

It was reported that not all patients have a clear goal or a help request themselves that is intrinsically motivated. Therefore, participants stressed the importance of identifying the patient's true motivation and whether this is intrinsic or extrinsic. Referral via the general practitioner was said to contribute to intrinsic motivation for some patients, as they viewed the referral as a wakeup call, whereas other patients lack intrinsic motivation and only visit the dietitian because their general practitioner told them to do so, according to participants.

### Deciding on the appropriate treatment approach

Participants highlighted that their treatment approach is shaped by the specific needs and expectations of patients. They reported that some patients primarily need knowledge and information on how to change their diet. In these cases, they reported to mainly act as information providers or educators, providing information on what to eat and why. Although information transfer is a standard component in every treatment, participants stressed that the emphasis differs per patient, as they have different knowledge and needs. An example given was that senior patients, in comparison to younger patients, frequently have had more experiences with losing weight and view weight loss more often as ‘going on a diet’, rather than a more holistic lifestyle change. On the other hand, participants observe that there is a group of patients who need the dietitian to act more as a motivational factor and counsellor. These patients do not only seek information but also rely on the dietitian for support, motivation and guidance. Participants gave account of differences in needs based on the patient's gender. In their experience, male patients more often benefit from a strong focus on practical instructions on diet and lifestyle changes compared to female patients, who generally require a stronger focus on emotional eating or other psychological factors behind their eating patterns.

Some people really see the consultations as a form of accountability, while others have more of a mindset like; ‘Well, what are you going to add in terms of information?’ (…) So, it also depends on what role they expect from me, I think. I even see someone now who wants to come in every week just to weigh themselves. I let them come in quickly for five minutes, and after three visits of five minutes each I declare a quarter‐hour session.

(Dietitian 7, interview)

Participants shared that many patients see them as their extrinsic motivator. They reported that this role is in particular helpful for patients with low health literacy. These patients often benefit from more frequent consultations in which the dietitian can monitor progress, (re)motivate them, and redirect if necessary. However, in the long term, patients need to build intrinsic motivation, according to participants. Participants also mentioned the value of a shared decision‐making approach which gives the patient the feeling of being equal and in a partnership. However, they also described situations in which they adopted a more directive approach based on patients' needs. For example, it was pointed out that senior patients may expect a more directive role from the dietitian, whereas younger patients prefer a collaborative decision‐making approach. In addition, participants perceived that patients with a non‐Dutch cultural background more often desire a more directive approach at the start compared to patients with a Dutch cultural background.

At first, they expect: you are the health care professional, you are the expert (…) before you try to form an equal relationship, it is important to make sure that they trust you. And in order to trust you (…) it is somewhat top‐down. (…) and only later you can get to the same level, step by step. (…) If I start with more interaction, so empowerment of the patient, I may create the idea that I am not enough of an expert.

(Dietitian 5, interview)

## THEME 3. SUPPORTING BEHAVIOUR CHANGE

Participants stressed that making dietary changes is a form of behaviour change. Therefore, it is essential to focus on supporting patients' behaviour change processes within dietetic treatment. Overall, they mentioned that successful treatment often is the result of a combination of facilitators at the patient level that enable them to start changing their behaviour. Figure 2 shows a matrix of patient factors that affect dietetic treatment.

[Interviewer: ‘What are the characteristics of the people who achieve weight loss compared to the rest?’] Participant: “A good education, intrinsic motivation, self‐management skills, health literacy skills, truly understanding the importance and having the means as well. I think the wallet does play a bigger role than I initially expected when I started [working as dietitian] (…). And especially recognising the importance and being willing to take the time [for the behavioural change process].”

(Dietitian 17, interview)

## ADDRESSING AWARENESS AND BELIEFS

Patients' beliefs in their capacity to change behaviour, known as ‘self‐efficacy’, was viewed as an important facilitator in behaviour change for some patients or as a barrier in others. Participants highlighted the value of further exploring the psychology behind the patient's behaviour. It was noted that this can be challenging, as not all patients are open to discussing psychology or simply do not expect this from a dietitian. Participants shared that asking the patient for permission to discuss topics around psychology or using exercises, such as conversation cards on values and beliefs, can be helpful to start the conversation. Participants also mentioned home assignments focusing on the relation between emotions and eating habits as examples to explore the origin behind behaviour. Discussing awareness of patients' preferences, behavioural patterns and underlying reasons and thoughts was regarded to be crucial for behaviour change. Another important patient factor reported was previous experience with dieting and dietetic treatment, as this can provide insights on what does and does not work for a patient. For patients with failed previous attempts, dietitians stressed the importance of focusing on building self‐esteem. Participants reported that patients with obesity more often than other patients struggle with low self‐esteem and feel judged within their environment, which may negatively impact their belief in themselves.

There are certain traits of people living with obesity, who have a lower self‐esteem, or more self‐judgement or are more troubled by the (views of) the outside world.

(Dietitian A, focus group discussion)

According to participants, it is therefore important to discuss topics around stigma, feelings of shame, and the difficulty of losing weight. They underlined the importance of examining one's own conscious or unconscious prejudices and beliefs around obesity to properly support patients with obesity. Furthermore, they accentuated the importance of discussing the origin of behaviour by talking about mental health and psychology. Participants stressed that although this is important, there is a limit to what they can discuss in their role as dietitians and that in some cases, they need to refer patients to a psychologist.

Sometimes, something else needs to happen, (then) people need to go work on themselves first. It is very confronting, but well. Sometimes you have someone in your practice who does not belong there (but with another health professional). There are clients who come here for their diet, because therein lies a problem, but behind the diet is an (psychological) issue that I cannot fix.

(Dietitian 12, interview)

### Attending to motivation and support

From the participants' perspective, motivation plays a crucial role in initiating and sustaining behaviour change, whereas successful behaviour change can, in turn, influence and enhance motivation. A patient's motivation was said to be very personal and could differ or evolve across the treatment process. Participants stressed that at the start, motivation is needed for patients to initiate the treatment and to make the first lifestyle changes. They shared that they use various techniques to identify patients' true motivation. Examples of these techniques include motivational interviewing and exercises designed to rate the motivation of the patient and discuss the patient's underlying motivation for change.

We were discussing how health is often not a real driving force for people. Of course, they consider it important, but when you delve deeper, it's not their true motivation (…) It's often like, “I actually want to fit into that dress, but it doesn't fit anymore”. You know, that's much more painful than health.

(Dietitian 9, interview)

Sociocultural background can play a role in patients' motivation according to participants.

I have the feeling that people with a non‐western background respond better to risk perception. So like: what could go wrong? And that people with a Dutch background respond better to what opportunities they have [to prevent this from happening].

(Dietitian 17, interview)

Participants reported that it can be effective to focus on examples of people in the patient's social environment who experienced positive or negative health outcomes related to their diet and discussing support from family and friends. They shared that encouraging the patient to share the weight loss process with others can help to create a more supportive social environment, especially for patients with a socio‐cultural environment in which social norms and support systems are more pronounced. In addition, participants reported that patients vary in how easily they ask for support. In that case, it can help to discuss the value of asking for support and expressing their support to patients, by giving compliments and showing empathy, which can also be very motivating for patients according to participants.

### Setting achievable goals

Setting realistic goals was reported to be highly important for successful behaviour change. Participants stressed the importance of assisting patients in setting realistic goals, including weight loss goals. Establishing realistic goals helps in maintaining patient motivation in the experience of participants. Furthermore, they underscored the importance of considering patients' individual factors in goal setting, taking patients' physical and mental capabilities into account. Participants agreed that patients with low health literacy benefit from a stronger focus on frequent evaluation, development of self‐management skills and bite‐size information transfer.

If someone has a bit of a lower educational level, then you advise small bite‐size steps that they can take. And then for the next consultation, decide on a clear agreement that suits them. If not, people will more often cancel the appointment.

(Dietitian 6, interview)

### Addressing barriers

Participants highlighted that for effective behaviour change, it is crucial to discuss patients' barriers in their behaviour change process and to find ways to deal with these.

I hope that they understand that it will happen, you will get setbacks. It will simply happen. It is not realistic to expect it not to happen. So what do you want to do with it? (…) The only thing what we can do about it is to reflect and learn from it: alright, where did this originate from? What happened here? And what can you do differently next time? So above all, some insight in how things occur.

(Dietitian 10, interview)

Barriers that were reported include financial stress, limited time, money or headspace for behaviour change at the patients' end. Other important barriers to successful treatment were a lack of sustained motivation and priority at the patient's end. Participants reported that it is important to address the value of dietetic consultation during times in which patients deal with such barriers. In their experience, patients who stop visiting the dietitian temporarily in busy times often do not return, resulting in premature dropout. Participants highlighted that unrealistic goals and a hyperfocus on achieving weight loss or other results are important barriers. These can be redressed by focusing on self‐compassion, setting achievable goals and focusing on the behaviour change process, rather than, for example, the number on the weighing scale. A final barrier to treatment effectivity that was pointed out is the maximum insurance coverage per year, resulting in (too) limited treatment time for some patients. Participants shared that they sometimes divide treatment time in more frequent short sessions to account for this limitation. However, this was often considered as insufficient for properly discuss a certain topic. Moreover, participants reported that some patients drop out when the insurance limit is reached and do not come back the next year. They noted that actively contacting patients when they cancel the next appointment may provide more insight into barriers to the continuation of treatment for patients and for dietitians to address these.

### Self‐monitoring

Participants mentioned that a focus on self‐monitoring with tools such as a diet‐tracking app is effective for patients with adequate digital skills to gain insight into their eating habits. On the other hand, diet diaries in a paper format were reported to be more appropriate for patients with low digital skills and health literacy. In addition, asking patients to take pictures of their food may help to complete a diet diary in patients with low literacy levels, according to participants.

For some people it works very well to, for example, fill in the diet tracker very committedly, that they really become conscious of: ‘this is what I eat every day and whether there is room for something extra or not’.

(Dietitian 3, interview)

Monitoring diet or behaviour can be a point of departure for discussing behaviour and causes of preferable and undesirable behaviour according to participants. When patients fail to perform self‐monitoring, reasons for this can be discussed as well, such as lack of motivation or confrontation with behaviour.

I can think of some clients who do not honour their commitments or say, ‘I was way too busy to keep such a food diary. Or to write down those emotions or…’ So there is also a bit of: ‘okay, what is the reason for that?’. I think that makes it more difficult to achieve goals.

(Dietitian 1, interview)

## THEME 4: PROVIDING ADVICE

Participants shared important insights on how they tailor their advice to patients' needs. This included ways to design suitable advice and choosing the best form of advice.

### Designing suitable advice

Participants stressed that for advice to be effective, it needs to be close to the patient's current behaviour to make the behavioural change as easy as possible. This is done by primarily suggesting small, easily incorporable adjustments to a patient's regular (eating) pattern.

But why does it work [the treatment] for one person and not for another, that is something I find challenging. However, I do think that the greatest successes are achieved by people who indeed, simply maintain a reasonably normal eating pattern, with relatively minor adjustments, and who are patient and approach it seriously.

(Dietitian 7, interview)

Participants named a sample menu that is close to the patient's normal diet, in combination with variation lists, information about healthy cooking and product knowledge as important basic components of their advice. These pieces of advice were seen as helpful for patients to work towards a healthy diet and vary between different options. Participants mentioned that for many patients, it is also important to discuss the setting in which they eat and provide advice related to the pace of eating, for instance, not to eat in front of a screen. In addition, participants stressed that diet is only one component of a healthy lifestyle and that it is important to pay attention to a wide range of other healthy lifestyle aspects, such as physical activity, sleep, stress, dealing with emotions and self‐care. In the treatment of patients with limited financial means, participants highlighted that advice should be tailored to their financial capabilities for the advice to be followed up effectively, by only including products that are affordable. Finally, participants shared that it is important to explain important underlying mechanisms in dietary change in an understandable way. An example of such a mechanism that was frequently mentioned is the nutrition–blood glucose relation for patients with diabetes.

### Choosing the form of advice

Participants highlighted that it is important to tailor the form in which they provide advice in a way that is understandable and practical for patients to use. They often share advice digitally but also on paper so that patients have a tangible copy somewhere in their house that they can more easily access and use to make notes. It was seen as important that information is practical, concrete and in simple language.

Participants shared that it is important to assess per individual patient what form of information transfer is needed for patients to gain knowledge or develop skills related to achieving a healthier lifestyle.

[Talking about patients with a low literacy level] Before I made entire pieces of advice with a lot of pictures of products that I cut out of the flyers from the super market. And then I thought, why not just go to the supermarket, and also do house visits.

(Dietitian B, Focus group discussion)

Supermarket tours, house visits and cooking workshops were mentioned as examples of ways to increase patients' product knowledge and cooking skills. The importance of using visuals and sound, rather than written text, was emphasised in the interviews. Visuals were said to be especially useful when explaining complicated mechanisms such as the aforementioned interplay between nutrition and blood sugar.

Visuals work for everyone, both highly educated as well as low educated.

(Dietitian 8, interview)

Visual information was considered to be particularly important in consultations with patients who do not have fluency in the Dutch language or who have a low literacy level. Examples that were given include showing videos or pictures rather than text and giving patients the option to take pictures or record audio when tracking their diet. Other strategies included working with interpreters or online translators for patients who are literate in another language. In addition, dietitians shared that for patients who have low proficiency in Dutch, it is helpful to have someone who can translate to accompany them.

## DISCUSSION

This study aimed to identify key components relevant for successful dietetic treatment of patients with obesity from the perspective of primary healthcare dietitians in the Netherlands. We found that dietitians perceive that the dietitian's capability to connect to patient needs is the key to successful treatment. Four themes were identified: building a good relationship, identifying patient needs, supporting behaviour change and providing advice.

A good interpersonal relationship was found to be the foundation for successful treatment and crucial for creating a trusted and open environment in which patients feel comfortable sharing personal information and their needs. A recent scoping review also reported that good dietitian–patient collaboration is the foundation for successful treatment of patients with chronic disease.^25^ The importance of a positive dietitian–patient relationship has been recognised in previous studies^26^, ^27^, ^28^ by both patients and dietitians, as well as other health professionals. A cross‐sectional analysis of medical treatment of HIV patients even found that ‘knowing the patient as a person’ was independently associated with receiving and adhering to therapy and positive health outcomes.^29^ A positive relationship is one of the components of delivering patient‐centred care (PCC).^30^ A study that evaluated PCC in dietetic practice in Australia found that one‐third of patients see different dietitians during treatment and highlighted the importance of consistent care by one dietitian to build a strong patient–dietitian relationship.^31^ However, our study found that when a good connection is lacking, it can be beneficial to refer the patient to another dietitian.

According to participating dietitians in the Netherlands, another key component of shaping treatment effectively was the dietitian's ability to identify patient needs and accordingly take on the role of an educator or counsellor. A similar distinction was noted in a study of nutrition treatment adherence, which found that an approach that is primarily informative was a barrier for some patients to continue with treatment. The authors noted that specifically patients with obesity and chronic diseases have a greater need for longer term treatment and benefit from a stronger focus on behaviour change counselling.^31^ Dietitians in our study indicated that many key components of successful dietetic treatment also apply to patients with overweight and other patient groups. However, an approach focused on counselling is indeed especially important in patients with obesity, with special focus on psychology, self‐esteem, feelings of shame and stigma. Accordingly, dietitians in our study highlighted the need to integrate psychological aspects in dietetic treatment. It is relevant that dietitians receive training in this area and refer patients with more significant psychological issues to another professional, such as a psychologist. Studies show that adding cognitive therapy to dietetic treatment is effective for long‐term success and less relapse in obesity.^32^ The shift from nutrition educator to nutrition counsellor has been recognised by other researchers,^33^, ^34^ and more emphasis has been placed on behaviour change counselling in dietetics education in the last decades.^35^ Tailoring the counselling approach based on patient characteristics such as motivation, health literacy and self‐efficacy was considered to be crucial for effective behaviour change. A systematic review of 35 obesity interventions indeed showed that motivation and self‐efficacy are important mediators for weight control.^36^ In addition, low health literacy is associated with obesity and specific nutrition skills such as estimation of portion size,^37^ understanding nutrition labels,^38^ searching for nutrition information and trust in information sources.^39^ A review of 33 studies on health literacy interventions found significant effects of using special education material on knowledge of patients with low health literacy and suggests using instruments to assess patients' health literacy. In this context, alignment with validated health literacy measures to assess the literacy levels of patients is important, as well as the use of readability assessment to match the literacy level of the target audience.^40^

A major theme that emerged in the interviews was the observation by dietitians that patients view them as their extrinsic motivator and supporter. Although dietitians acknowledged that extrinsic motivation can be effective, they emphasised that, in their experience, focusing on increasing the patient's intrinsic motivation is essential in the path to a successful treatment. This aligns with other studies that stress the importance of intrinsic motivation.^34^, ^41^, ^42^ Previous literature has shown that support from the dietitian is one of the most important facilitators of successful weight loss programmes.^43^, ^44^, ^45^, ^46^ In this, various forms of support are mentioned, including regular support, such as face‐to‐face consultation, and support in the form of guidance and accountability to the dietitian. Together, support can significantly improve treatment adherence.^47^ Furthermore, dietitians recognised the importance of helping patients address barriers they encounter by setting realistic goals, focusing on motivation and self‐care. They noted that these barriers do not only influence treatment outcomes but also the decision to (dis)continue treatment. By recognising and discussing possible obstacles, dietitians may offer timely support to help patients overcome these challenges and continue their treatment, ultimately improving patient outcomes, as treatment time is related to greater weight loss.^48^ Dietitians indicated that, from their perspective, individual patient factors often play a significant role in determining the success of the treatment and related treatment outcomes. However, as dietitians differ in their personal approach and abilities to tailor their treatment to individual patient factors and needs, patients may achieve different treatment outcomes with different treatment approaches.

It is important to note that the insights of our study reflect perspectives and experiences from the viewpoint of (Dutch) dietitians. It is equally important to investigate and incorporate the patients' viewpoints in research to ensure a comprehensive understanding of the factors influencing treatment success.

## STRENGTHS AND LIMITATIONS

This study has multiple strengths. First, two different data collection methods were used. The interviews allowed us to explore individual views on the topics in depth. These insights then shaped the focus group discussions, which focused on key themes from the interviews. Additionally, the focus group discussions were valuable because it provided participants the opportunity to build on each other's ideas. This collaborative environment resulted in broader and more dynamic conversations than we could have achieved in individual interviews alone. Moreover, the focus group discussions helped to gain insights on where participants agreed and disagreed on various topics. These differences were, for example, influenced by factors such as years of practice and the way in which different dietitians were educated. Second, the diverse backgrounds of the participating dietitians provided a broad spectrum of perspectives; the participants varied in age, geographic area of residence, training backgrounds from different universities of applied sciences and a range of professional experiences. Third, dietitians were selected based on their experience with patients varying in characteristics, leading to an exploration of important treatment components for various patient factors. During purposive sampling, special attention was given to include dietitians who treat patients with low and limited health literacy levels, as this patient group is often under‐represented in research. Finally, although dietetic practice varies across countries, we believe that our results are relevant in a wider context and provide valuable insights on how to deliver treatment tailored to patient needs.

The study also has some limitations. Participants reported that they do not often treat patients with low literacy levels, which might have led to limited insights into crucial components of the treatment of patients with low literacy levels. Furthermore, participants in our study often do not stay in contact with patients who dropped out; therefore, they could not report on patients' reasons for dropping out. Consequently, it was difficult to arrive at conclusions related to long‐term treatment (in)effectivity.

### Implications for practice and research

This study explored dietitians' perspectives on key components for dietetic treatment of patients with obesity. Future research is essential to confirm whether these perspectives indeed reflect components that affect the outcomes of dietetic treatment. Additionally, the study results suggest that there is a need for further investigation into the relationship between patients' individual characteristics and different counselling strategies. More quantitative data on these strategies are required to gain insight into their effect on both weight loss and the experiences of patients with different characteristics. Moreover, this study confirms the importance of incorporating counselling skills focused on promoting self‐efficacy, motivation and behavioural change in educational curricula and professional platforms for dietitians. Dietetic education and guidance should include strategies for choosing from the wide variety of treatment components, based on individual patient needs. The results of this research can be used in the subsequent phase of the research project Dietetics building the future, in which important components in dietetic treatment will be translated into practical tools for dietitians to use in their daily practice. Additionally, interviews with patients living with overweight and obesity will be conducted in the next phases. Their experiences are crucial to gain deeper insights into the dietetic treatment experienced by patients and will inform the development of effective tools and strategies.

## CONCLUSION

This study demonstrates that dietitians perceive the ability to build a trusted relationship, in which patient needs are properly explored and addressed, as the key to successful dietetic treatment of adults with obesity. From the perspectives and experiences of dietitians, four themes were identified: building a good relationship, identifying patients' needs, supporting behaviour change and providing advice. The dietitian's ability to build a trusted relationship in which patients' needs are properly explored and responded to is essential for successful treatment of patients with obesity. Important treatment components include adjusting the treatment approach to patients' self‐efficacy, motivation and health literacy, and taking on the role of a counsellor to support behaviour change.

## The Dietetics Building the Future consortium members

**Kirsten Berk** (Erasmus Medical Centre, Department of Internal Medicine, division of Dietetics, Rotterdam, The Netherlands), **Harriët Jager‐Wittenaar** (Hanze University of Applied Sciences, Research Group Healthy Ageing, Allied Health Care and Nursing, Groningen, The Netherlands; Radboud university medical center, Department of Gastroenterology and Hepatology, Dietetics, Nijmegen, The Netherlands; Vrije Universiteit Brussel, Faculty of Physical Education and Physiotherapy, Department Physiotherapy and Human Anatomy, Research Unit Experimental Anatomy, Brussels, Belgium), **Hinke Kruizenga** (Amsterdam UMC, Department of Nutrition and Dietetics, Amsterdam, The Netherlands; Amsterdam Movement Sciences, Ageing & Vitality, Amsterdam, The Netherlands), **Jacqueline Langius** (The Hague University of Applied Sciences, Faculty of Health, Nutrition and Sport, Department of Nutrition and Dietetics and Research group Rehabilitation and Technology, The Hague, The Netherlands), **Barbara van der Meij** (Wageningen University and Research, Human Nutrition and Health, Research group Global Nutrition, Wageningen, the Netherlands; HAN University of Applied Sciences, Research Group Nutrition, Dietetics, and Lifestyle, Nijmegen, The Netherlands), **Elke Naumann** (HAN University of Applied Sciences, Research Group Nutrition, Dietetics, and Lifestyle, Nijmegen, The Netherlands), **Marieke Plas** (Dutch Association of Dietitians, Amersfoort, The Netherlands), **Annemieke van de Riet** (Wageningen University and Research, Human Nutrition and Health, Research group Global Nutrition, Wageningen, the Netherlands), Marian de van der Schueren (Wageningen University and Research, Human Nutrition and Health, Research group Global Nutrition, Wageningen, the Netherlands; HAN University of Applied Sciences, Research Group Nutrition, Dietetics, and Lifestyle, Nijmegen, The Netherlands).

## AUTHOR CONTRIBUTIONS

**Annemieke van de Riet**: Conceptualisation; methodology; data collection and analysis; writing—original draft; review and editing and project administration. **Rebecca S. Otte:** Conceptualisation; methodology; data collection and analysis; writing—original draft; review and editing and project administration. **Harriët Jager‐Wittenaar**: Conceptualisation; methodology; supervision and critical review of manuscript. **Marian A. E. de van der Schueren**: Conceptualisation; methodology and critical review of manuscript. **Elke Naumann**: Conceptualisation; methodology; supervision and critical review of manuscript. All authors read and approved the final manuscript.

## CONFLICT OF INTEREST STATEMENT

The authors declare no conflict of interest.

## ETHICS STATEMENT

Ethical approval specifically for this qualitative study was granted by the Research Ethics Committee of Wageningen University and Research (WUR‐REC) under approval number 2023‐034. The Committee concluded that the research deals with ethical issues in a satisfactory way and that it complies with the Dutch ‘Code of Ethics’ for research in the social and behavioural sciences involving human participants.

### PEER REVIEW

The peer‐review history for this article is available at http://www.webofscience.com/api/gateway/wos/peer-review/10.1111/jhn.13387.

## Supporting information

Supplementary Information

## Data Availability

The data that support the findings of this study are available on request from the corresponding author. The data are not publicly available due to privacy or ethical restrictions.
